# Size Distribution Analysis with On-Chip Multi-Imaging Cell Sorter for Unlabeled Identification of Circulating Tumor Cells in Blood

**DOI:** 10.3390/mi10020154

**Published:** 2019-02-25

**Authors:** Masao Odaka, Hyonchol Kim, Yoshiyasu Nakamura, Akihiro Hattori, Kenji Matsuura, Moe Iwamura, Yohei Miyagi, Kenji Yasuda

**Affiliations:** 1 Organization for University Research Initiatives, Waseda University, 3-14-9 Okubo, Shinjuku, Tokyo 169-0072, Japan; masao_odaka@aoni.waseda.jp (M.O.); a.hattori@aoni.waseda.jp (A.H.); k.matsuura@aoni.waseda.jp (K.M.); 2 Waseda Bioscience Research Institute in Singapore (WABOIS), Helios, 11 Biopolis Way, Singapore 138667, Singapore; 3 Biomedical Research Institute, Advanced Industrial Science and Technology (AIST), 1-1-1 Higashi, Tsukuba, Ibaraki 305-8566, Japan; kim-hc@aist.go.jp; 4 Molecular Pathology and Genetics Division, Kanagawa Cancer Center Research Institute, 1-1-2 Nakao, Asahi-ku, Yokohama 241-0815, Japan; nakayosi@gancen.asahi.yokohama.jp (Y.N.); miyagi@gancen.asahi.yokohama.jp (Y.M.); 5 Department of Pure and Applied Physics, Graduate School of Advanced Science and Engineering, Waseda University, 3-4-1 Okubo, Shinjuku, Tokyo 169-8555, Japan; iwme523o@moegi.waseda.jp; 6 Department of Physics, School of Advanced Science and Engineering, Waseda University, 3-4-1 Okubo, Shinjuku, Tokyo 169-8555, Japan

**Keywords:** imaging biomarker, circulating tumor cell (CTC), multi-imaging cell sorter, cell cluster, size distribution analysis

## Abstract

We report a change of the imaging biomarker distribution of circulating tumor cell (CTC) clusters in blood over time using an on-chip multi-imaging flow cytometry system, which can obtain morphometric parameters of cells and those clusters, such as cell number, perimeter, total cross-sectional area, aspect ratio, number of nuclei, and size of nuclei, as “imaging biomarkers”. Both bright-field (BF) and fluorescent (FL) images were acquired at 200 frames per second and analyzed within the intervals for real-time cell sorting. A green fluorescent protein-transfected prostate cancer cell line (MAT-LyLu-GFP) was implanted into Copenhagen rats, and the blood samples of these rats were collected 2 to 11 days later and measured using the system. The results showed that cells having BF area of 90 μm^2^ or larger increased in number seven days after the cancer cell implantation, which was specifically detected as a shift of the cell size distribution for blood samples of implanted rats, in comparison with that for control blood. All cells with BF area of 150 μm^2^ or larger were arranged in cell clusters composed of at least two cells, as confirmed by FL nucleus number and area measurements, and they constituted more than 1% of all white blood cells. These results indicate that the mapping of cell size distribution is useful for identifying an increase of irregular cells such as cell clusters in blood, and show that CTC clusters become more abundant in blood over time after malignant tumor formation. The results also reveal that a blood sample of only 50 μL is sufficient to acquire a stable size distribution map of all blood cells to predict the presence of CTC clusters.

## 1. Introduction

Circulating tumor cells (CTCs) are considered to play a major role in the formation of additional metastatic tumors as seeds for subsequent tumor growth [1,2,3]. Reliable technologies for the quantitative detection of CTCs in blood [4,5,6,7,8] have the potential to achieve minimally invasive cancer diagnostics, called liquid biopsies, in contrast to conventional biopsies.

One of the most common approaches for finding target cells is to use molecules specific to such cells, namely, molecular biomarkers, on the cell surface [1,3,6,9,10]. However, this approach sometimes produces false-negative results because of the variety of molecular expression of the targeted cells. For example, when aiming to identify the epithelial–mesenchymal transition (EMT) and its intermediate states in human disease, as crucial drivers of organ fibrosis and tumor progression, there is a need for caution when performing phenotypic identification using molecular biomarkers [11,12].

Because of the functional limitations of conventional molecular biomarker approaches, other physical characteristics such as the size of cells [13,14] or biophysical characteristics such as the difference of affinity of cell surfaces [15,16,17]. In our previous studies, we proposed an original approach for CTC detection in blood based on analyses of the morphometric parameters of cells without any antibody staining, with these parameters acting as “imaging biomarkers” [18,19]. For this approach, we developed a high-speed cell image recognition and analysis system to identify the target cells using their morphological characteristics during their flow within a narrow microfluidic pathway for sorting. We also improved this system and developed an on-chip multi-imaging flow cytometry system [20,21], which allows the simultaneous acquisition of both bright-field (BF) and fluorescent (FL) images when scanning all cells in blood. Using this system, clustered CTCs were specifically detected for identifying not only their cluster sizes but also their nucleus shapes and numbers within clusters simultaneously in the blood of cancer cell-implanted rats.

Combining this powerful complementary pair of imaging biomarkers, BF and FL images, for single cells is one option for identifying CTCs without the need for antibody labeling. For example, larger cellular sizes are thought to be indicative of tumor cells [22,23,24,25], and a larger nucleus and multiple nuclei in single cells are also morphometric phenotypes of cancer cells [26,27,28,29,30,31,32]. Hence, the search for target cells using the combination of two imaging biomarkers simultaneously, especially using cell size and nucleus conformation, should be useful for identifying tumor cells without antibody staining. In particular, as it is difficult to distinguish larger single cells and clustered cells by diffraction detection, this combination of two imaging biomarkers is a simple but powerful alternative approach for CTC cluster identification [20].

Numerous cellular analytical technologies using microfluidic devices have been developed recently, for both biological and medical diagnoses [7,24,33,34,35,36,37,38]. Recently, the change of morphological characteristics of cells as they pass through the cell cycle was used for sorting of particular yeasts in a fully automated low-cost imaging cell sorter with a cell detection rate of 5 s [39]. A low-cost microfluidic eight-way simultaneous image data-based sorting system mounted on a fluorescence microscope was also demonstrated with a sorting rate of 0.5 s resolution [40]. A deep-learning-assisted imaging cell sorter system has also been reported [41], in which the abilities of image analysis and cell identification were significantly improved, while maintaining a time resolution on the sub-second order.

Previous reports on CTC detection suggested that CTCs may show a tendency to form clusters [7,20]. However, clear evidence of this has not yet been presented and, to the best of our knowledge, there have been no quantitative studies on the identification of clustered cells in the blood, such as on their frequency among blood cells and their relationship with the progression of metastatic cancer. To estimate the size of the population of such cells in the blood, we need to measure the total number of all of those cells over time during the progression of metastatic cancer.

In this study, as a follow-up to our earlier experiment [20], an on-chip multi-imaging cell sorting system was applied to measure the changes in cell size, number of cell clusters, and their distribution over time after a green fluorescent protein (GFP)-transfected prostate cancer cell line (MAT-LyLu-GFP) had been implanted into rats. Acquisition of the change over time of the distribution of imaging biomarkers in whole positive and control blood samples accompanying the progression of metastasis cancer should also provide insights about a possible or desirable index of CTC capture.

## 2. Materials and Methods

### 2.1. On-Chip Multi-Imaging Flow Cytometry System with Agarose Gel Electrodes

**Figure 1:**
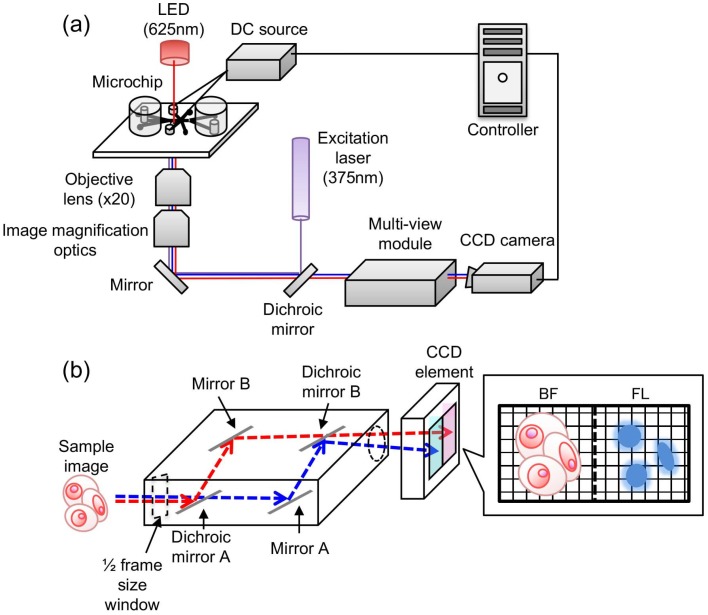
Set-up of the on-chip multi-imaging cell sorter system. (**a**) summary of the on-chip multi-imaging flow cytometry system. The system was composed of eight major modules: (i) microfluidic chip, (ii) bright-field (BF) light source, (iii) fluorescent (FL) light source, (iv) objective lens with image magnification optics for depth of field extension, (v) multi-view unit, (vi) high-speed charge-coupled device (CCD) camera, (vii) sorting DC voltage source, and (viii) controller, as numbered in the figure; (**b**) schematic of the multi-view module.

Figure 1a shows a schematic image of the on-chip multi-imaging flow cytometry system improved from our previous system [20], which was composed of the following eight major modules: (i) microfluidic chip, (ii) LED bright-field (BF) light source (625-nm wavelength), (iii) fluorescent (FL) excitation light source (375 nm for Hoechst 33342 reversible staining of nuclei), (iv) objective lens with image magnification optics for depth of field extension, (v) multi-view unit, (vi) high-speed digital charge-coupled device (CCD) camera, (vii) sorting DC voltage pulse source, and (viii) image analysis and sorting controller (Figure 1a). The major changes from our previous system are as follows: (iii) FL excitation light source and (iv) objective lens with extended depth of field. To reduce the loss of fluorescence intensity, we reduced the five dichroic mirrors for multi-fluorescence detection prior to the multi-view module to one dichroic mirror for a single fluorescent light source. For the extension of the depth of field of cell images in the microfluidic pathway, we also added the image magnification optics to the objective lens having 20× magnification and a 0.75 numerical aperture [42].

Figure 1b shows the simultaneous BF and FL image acquisition pathway in the multi-view unit [21,43]. In brief, sample images acquired with 1/2 CCD capture component size are separated with dichroic mirror A and introduced into the optical paths of BF (red dashed line) and FL (blue dashed line) lights. The angles of mirrors A and B are adjusted to place BF and FL images onto each half of the CCD capture component and two images are simultaneously acquired on the CCD camera. The same captured cell images of BF and FL wavelengths are compared to enable estimation of the number and size of nuclei in each cell with BF position matching of cells in the image analysis.

After the setting of suitable thresholds for the imaging biomarker parameters, feedback signals were sent to the sorting module. In addition, DC pulse voltages (typically 40 V and 100 μs in length) were applied to sort target cells flowing in the microchannel of the chip through the pair of agarose gel electrodes in the microfluidic chip to shift the course of flow of the cells. Finally, this shifting led them to be introduced into the collection channel instead of the discarding channel.

### 2.2. Microfluidic Chip

**Figure 2:**
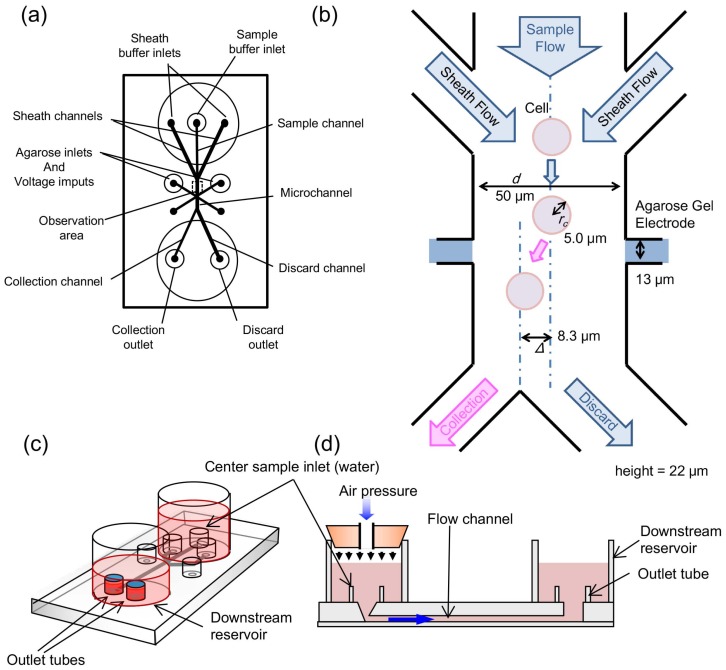
Three-inlet two-outlet agarose gel electrode microfluidic chip design. (**a**) schematic image of the top of the chip (same design as we reported in a previous paper [20]); (**b**) schematic drawing of the cell analysis and site of separation of the microfluidic pathway; (**c**) bird’s eye view of the whole chip design, and (**d**) a cross-sectional image of the chip.

A disposable microfluidic chip was fabricated with poly(dimethylsiloxane) (PDMS, SYLGARD 184 silicon elastomer; Dow Corning Co., Midland, MI, USA) attached to a thin glass slide by the same procedure as we reported previously [20], and was used for monitoring and sorting of cells. The designs of microfluidic flow pathways and agarose gel electrodes in the chip were also kept the same as in the chip in the previous report to maintain the consistency of the procedures [20] (Figure 2a). As shown in Figure 2a,b, the upper stream was divided into three channels; the central one was used for the sample inlet and the remaining two side channels were used for sheath buffer inlets. After the meeting of sample and sheath flows, the width of the sample flow was focused by hydrodynamic focusing, which allowed imaging of each single cell upon the arrangement of all of the cells in a straight line. The hydrodynamic focusing in this chip design conferred several advantages for cell sorting, such as (1) centering the samples in the microfluidic flow, (2) adding spaces between neighboring samples when lining them up, and (3) aligning the orientation of samples in the direction of flow. Advantage (2) in particular contributed to the separation of neighboring cells with enough additional space to be able to distinguish cell clusters from gathering isolated cells.

### 2.3. Agarose Gel Electrodes for Cell Sorting

Agarose gel electrodes have several advantages compared with metal electrode arrays. For example, they can generate ion currents and hence prevent bubble formation from electrolysis in the microfluidic flow, even when higher voltages are applied. Our agarose gel electrode cell sorting in a microfluidic chip can be briefly described as follows (Figure 2b) [18,20,44]: (i) a direct ion current pulse is generated by applying a pulsed DC voltage between a pair of agarose gel electrodes, which are designed to contact two side walls of a microfluidic flow channel when a target cell is recognized; (ii) the position of the target cells is shifted from the center of the microfluidic flow due to the electrophoretic force; and (iii) the target cells flow into the collection outlet. In this system, cells were applied to a sample inlet and introduced into the observation area of the microchannel (Figure 2b–d). The flow speed was controlled by the air pressure of the upper stream. The channel width (*d*) and height were 50 μm and 22 μm, respectively, and the position of cells flowing from the sample inlet was set on the channel center with hydrodynamic focusing of the two side sheath flows. The downstream part of the chip was divided into two channels: a “discard” outlet and a “collection” outlet. When the cells were shifted more than 8.3 μm (Δ) from the center of the channel, cells flowed into the “collection” outlet. Otherwise, all cells were transported to the “discard” outlet. Once a direct current voltage was applied to the agarose gel electrodes (13 μm in width and 22 μm in height), the cells were shifted in the direction of the collection outlet branch by an electrophoretic force.

### 2.4. Imaging Biomarker Detection

As described in detail in our previous report [20], we identified target cells by measuring the imaging biomarkers of cells. After removing a prerecorded background image, 8-bit (256-step) grayscale values of obtained cell images were transformed into binary images using threshold values based on the average intensities of BF and FL images of each cell. In this study, we used the same analysis algorithm as in our previous report [20], and acquired cell area, actual perimeter, number of cells in a cluster, and aspect ratio from the BF image, as well as nucleus area and number of nuclei in each cell from the FL image, with reference to the BF contour images of cells.

These calculations of imaging biomarkers were performed within an interval of 200 fps in the image analysis and sorting controller module. In this study, manual calculations of the imaging biomarkers, including a few modifications for apparently failed auto-calculations caused by the failure of continuous detection of the cell number, cell perimeter, and nuclear number and contour identification procedures, were performed as post-processing to confirm the reliability of the obtained imaging biomarker values. To confirm the performance of this system, we set those cells that exceeded the BF area threshold of 300 μm^2^ as target cells. We collected these target cells in the target collection reservoir, in line with our previous report [20]. For the analysis of each 50-μL sample of blood, approximately 3 h was required to acquire both the images and the imaging biomarker values of the cells.

### 2.5. Preparation of Sample Blood

This study was carried out in strict accordance with the Act on Welfare and Management of Animals of the Ministry of the Environment, Japan. The protocol was approved by the Animal Experiment Committee of Kanagawa Cancer Center (permit number 21-02). MAT-LyLu, a rat prostate cancer cell line established from the original Dunning R3327 tumor [45], was a generous gift from the original founders through Dr. Hisao Ekimoto, at the Oncology Section, Laboratory of Biology, Nippon Kayaku Co., Ltd. Tokyo, Japan, and was maintained.

To maintain the consistency of results in the experiments, all of the procedures of sample preparation were the same as in our previous study [20]. One difference from this former study was GFP labeling of MAT-LyLu cells for distinguishing MAT-LyLu from other blood cells. MAT-LyLu-GFP was established from MAT-LyLu, in which the GFP gene was transfected using a lentiviral transfection system. The MAT-LyLu-GFP was adjusted to a concentration of $5\times 10^{6}$ cells in 200 μL of cell culture medium (RPMI 1640; Life Technologies Co., Grand Island, NY, USA) and implanted into dorsal subcutaneous tissue of Copenhagen rats (males, 6 weeks old). Two days after implantation, 100 μL of blood from each of six rats was collected from the subclavian vein using a collection tube containing heparin. As controls, either the cell culture medium (Control 1) or a human ovary cancer cell line, ES-2 (Control 2), was implanted into three individuals each, and the blood was collected in the same manner as described above. Collected blood samples were hemolyzed on the same day without cell fixation using commercial reagent (BD Pharm Lyse; BD Biosciences, San Jose, CA, USA) for 10 min, washed by centrifugation, resuspended in phosphate-buffered saline (PBS) containing 10 mg/mL bovine serum albumin (BSA) and 100 ng/mL Hoechst 33342 (Dojindo Laboratories, Kumamoto, Japan), and incubated for 10 min to stain the nuclei. Each sample was then washed again by centrifugation, suspended in 5% glucose containing 2 mg/mL DNase I (Roche Diagnostics K.K., Basel, Switzerland), and applied to the sample inlet on a microchip. To observe the change over time of the population ratio of imaging biomarkers, 100-μL blood samples were also acquired from the same 12 rats 4, 7, 9, and 11 days after the implantation in the same manner as described above and measured.

### 2.6. Procedure of Imaging Flow Cytometry

The blood samples were applied to the sample inlet of the system with a sample volume of 50 μL. The cell suspension (i.e., 5% glucose) was used for the sheath buffer. Air pressure was applied to both sample and sheath buffer inlets simultaneously using a syringe pump to control the flow speed of samples (Figure 2c,d). In this system, multi-imaging BF and FL observations of sample blood having flow velocity of 3 mm/s with the application of air pressure of 1 kPa were performed with an acquisition rate of 200 frames per second (fps) through the multi-view unit. The acquisition rate can be accelerated up to 5000 fps by switching the image analysis from the software-based processing module to the field programmable gate array (FPGA)-based processing module; however, the intensities of FL images are the decision parameter for optimizing the maximum acquisition rate and flow velocity for practical use [46].

## 3. Results and Discussion

### 3.1. Detection of Time-Course Change of Imaging Biomarkers of Cancer-Implanted Rat Blood

In our previous study on CTC cluster detection [20], cell clusters were specifically observed in cancer cell-implanted blood. To evaluate this observation, a rat prostate cancer cell line in which GFP was transfected, MAT-LyLu-GFP, was implanted into Copenhagen rats. The blood of these rats (referred to as “positive blood” hereafter) was collected over time from 2 days (Day 2) until 11 days (Day 11) after the implantation, and the change over time of the imaging biomarker distributions of cells in the blood was measured using our system. As controls, two kinds of rat blood were also measured in the same manner: one with only culture medium injected (control 1) and the other with implantation of a human ovary cancer cell line, ES-2 (control 2). The blood of six positive cases and three cases from each of two controls was collected from the rats.

**Figure 3:**
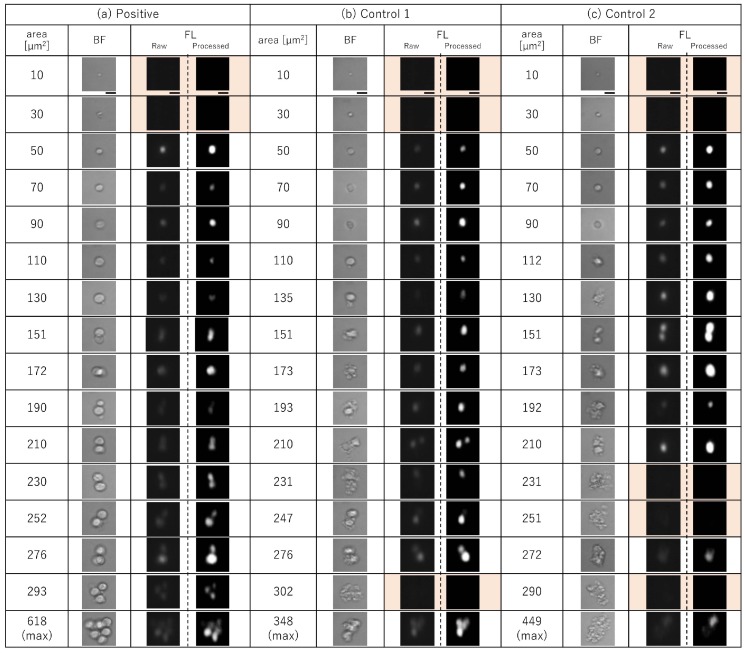
Typical cell images for positive (**a**), control 1 (**b**), and control 2 (**c**) blood having different BF cell areas at 20-μm^2^ intervals at 11 days after implantation. (left) BF; center, raw FL; and (right) processed FL images. Orange hatched frames in FL images, no nucleus in cells, that is, debris. Bars, 10 μm.

Figure 3 shows typical cell images acquired by our on-chip multi-imaging cell sorter system for positive (Figure 3a), control 1 (Figure 3b), and control 2 (Figure 3c) blood. This blood had various BF cell areas at 20-μm^2^ intervals from 10 μm^2^ at 11 days after implantation. For precise confirmation of the configuration of cells to distinguish them from debris in the samples, FL images (i.e., configurations of cell nuclei) were analyzed, as shown in Figure 3. The intensities of raw FL images were an order of magnitude weaker than the BF images because we optimized the sensitivity of the CCD device for BF images of half the CCD detection area to maximize the 8-bit, 256-step grayscale BF contrast of those cells; under this setting of CCD device sensitivity, the peak of raw FL image intensities could be seen to be darker and of much lower intensity than the peak of 256 steps. However, as shown in the processed FL images in Figure 3, the recorded FL intensities were much higher than for lower 4-bit scales (lower 16 steps in a total of 256 steps of grayscale), which was sufficient for recognition as nuclei by the digitized peak recognition processing procedures after removing a prerecorded background image.

These paired BF and FL images indicate one of the advantages of this multi-imaging simultaneous single CCD device recording. For the position matching of FL images of nuclei with BF cell images, those two images were acquired simultaneously on each half of the CCD detector, and the locations of sensor unit pixels were geometrically consistent with each other. Hence, the images of the FL nucleus should exist at just the same position in BL cell images. This entire matching process was carried out in one CCD-image analysis procedure. Hence, we can distinguish whether each cell has a single nucleus, multiple nuclei, or no nucleus at all. If we use two CCD cameras for BF and FL images, more digital processing procedures are needed.

For example, as shown in the orange hatched frames in Figure 3, none of the particles smaller than 30 μm^2^ in blood had any FL-stained area. Hence, those particles appeared to be microvesicles or large oncosomes. It can also be applied for the identification of debris in larger cell area samples. For example, in positive samples (Figure 3a), even in larger cell clusters, the same numbers of nuclei were observed at the right position of each cell in BF images. However, in both Control 1 and Control 2 (Figure 3b,c, respectively), the estimated number of cells and number of nuclei did not match. Most larger clusters had no nucleus or a single nucleus. This indicates that the larger clusters in Control 1 and 2 were mostly composed of debris.

These images also showed the effectiveness of hydrodynamic focusing for the separation and spacing of each sample. As shown in BF images, each cell or cell cluster was separated and isolated sufficiently to avoid misjudging multiple neighboring single cells as clusters.

### 3.2. Time-Course Change of Cell Area

**Figure 4:**
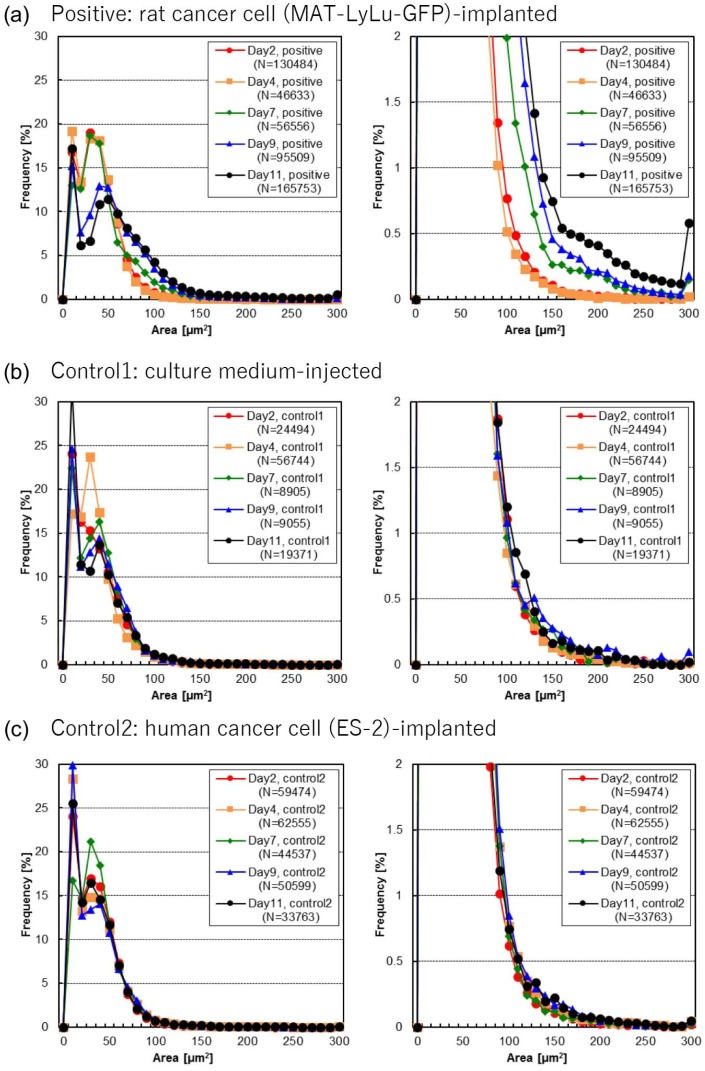
Size distribution of BF cell areas in positive (**a**), control 1 (**b**), and control 2 (**c**) blood. Each size distribution map indicates 2, 4, 7, 9, or 11 days after the implantation of rat cancer cells (**a**, positive), culture medium (**b**, control 1), or human cancer cells (**c**, control 2). Measurements were performed for six rats for positive cases and three for controls. The size distribution maps on the right are magnified versions of those on the left, to emphasize the detailed findings for large cells. Here, N represents the total number of cells in each size distribution map.

Figure 4 shows the size distribution of BF cell area in blood 2, 4, 7, 9, and 11 days after implantation for positive (Figure 4a), control 1 (Figure 4b), and control 2 blood (Figure 4c). The left graphs present the whole size frequency distributions, while the right ones are magnified graphs enabling observation of the increase of larger cells in more detail.

In Figure 4, several peaks were observed in the size distributions. The BF and FL images of those samples shown in Figure 3 revealed the following characteristics: (i) Cells having a BF area of approximately 10 to 30 μm^2^ were observed. They formed a major peak in the size distribution of cells in all three samples and represented the remains or debris of single red blood cells. (ii) Cells having a BF area of approximately 50 to 70 μm^2^ formed another major peak in the size distribution in all three samples, representing single white blood cells. (iii) Cells having a BF area of approximately 90 to 130 μm^2^ were found specifically in Mat-LyLu-GFP cancer cell-implanted positive blood. They increased their population and size over time, which was detected as a shift of the peak in the distribution map of BF cell area. These represented either single large cells or a combination of a cell with small debris in positive blood, and only the combination of a cell with small debris in controls. (iv) Seven days after the implantation, another minor peak corresponding to a much larger size appeared at approximately 150 to 200 μm^2^ only in Mat-LyLu-implanted positive blood (Figure 4a) and gradually increased as more than twice in 11 days after the implantation. All cells having an area larger than 150 μm^2^ were in the form of cell clusters, composed of at least two cells. (v) Large cell clusters composed of three or more cells, having BF areas of 270 μm^2^ or larger, were specific to positive blood, while those in controls were combinations of cells and large debris (Figure 3b,c). In contrast, (vi) the formation of a peak corresponding to 90 to 130 μm^2^ was not observed in either of the two controls (Figure 4b,c).

As described above, large cells having a BF area of approximately 90 to 130 μm^2^ were specifically observed in positive blood, and notably increased in number over time along with cell clusters (Figure 4a). One possible explanation for this is that these cells are single CTCs. In accordance with the result in Figure 4a, one possible assumption about the CTC formation in this study is that, first, CTC clusters appeared in blood at about seven days after malignant tumor formation, and second, single CTCs drastically increased in number at 9 to 11 days after tumor formation, along with an increase of CTC clusters.

**Figure 5:**
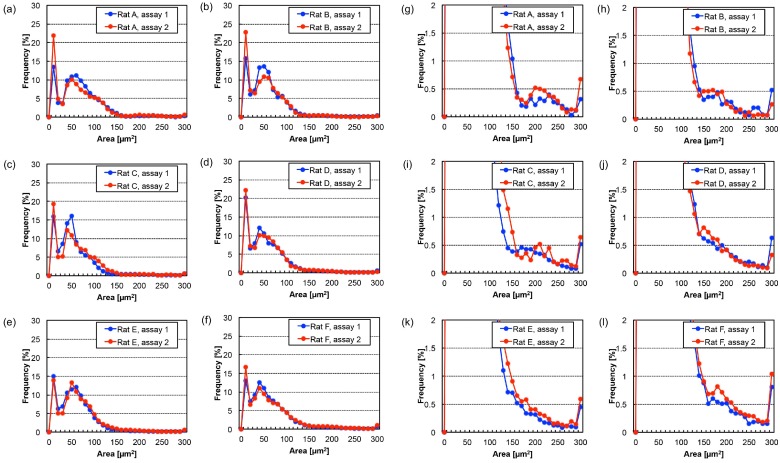
Size distribution of BF cell areas in positive blood from six individual rats 11 days after cancer cell implantation. (**a**–**f**) indicate the results for six rats with overlaps of assays performed twice using the same blood samples, to confirm the reproducibility of the measurements; (**g**–**l**) are magnified versions of (**a**–**f**) to emphasize the frequency values in large cell areas (e.g., (**g**) is a magnified version of (**a**)).

These measurements were repeated at least twice for the 50-μL blood samples; their size distribution maps showed the same tendencies. For example, as shown in Figure 5, analyses of two 50-μL blood samples of Day 11 positive blood from all six rats indicated that a blood sample volume of 50 μL was sufficient to acquire a stable size distribution map, allowing recognition of the increase in CTCs in blood by watching the change of their size distribution map. This was particularly the case for cells having a BF area of around 90 to 130 μm^2^, 150 to 200 μm^2^, and 270 μm^2^ or larger.

The results in Figure 3 and Figure 4 also indicate that (i) the formation of large cell clusters composed of three or more cells was specific to positive blood, (ii) the formation of cell clusters started seven days after the cancer cell implantation in positive blood, detected as the formation of a minor peak in the size distribution of BF area as shown in Figure 4a, and (iii) single large cells clearly increased in number at Day 9 and Day 11 in positive blood. This was also detected as an increase of intermediate BF area between major and minor peaks in the size distribution shown in Figure 4a. Result (i) is also consistent with our previous study [20], and in accordance with the conclusion in that previous study, these cell clusters were expected to be CTCs.

As shown in Figure 4b,c, some of the cells in controls were also detected as cell clusters having a large BF area, although these were false-positive results (i.e., combination of cells with small or large debris, Figure 3b,c). The discrimination of true cell clusters from false-positives is in principle difficult when counting only the cells having a BF area larger than a certain threshold. In contrast, the increase of true cell clusters was clearly detected by observing the change in the BF area size distribution over time, as shown in Figure 4a. This indicates that monitoring the distribution shape is useful for reliably detecting irregular cells such as cell clusters in blood.

### 3.3. Time-Course Change of Cell and Cell Cluster Perimeter Lengths

**Figure 6:**
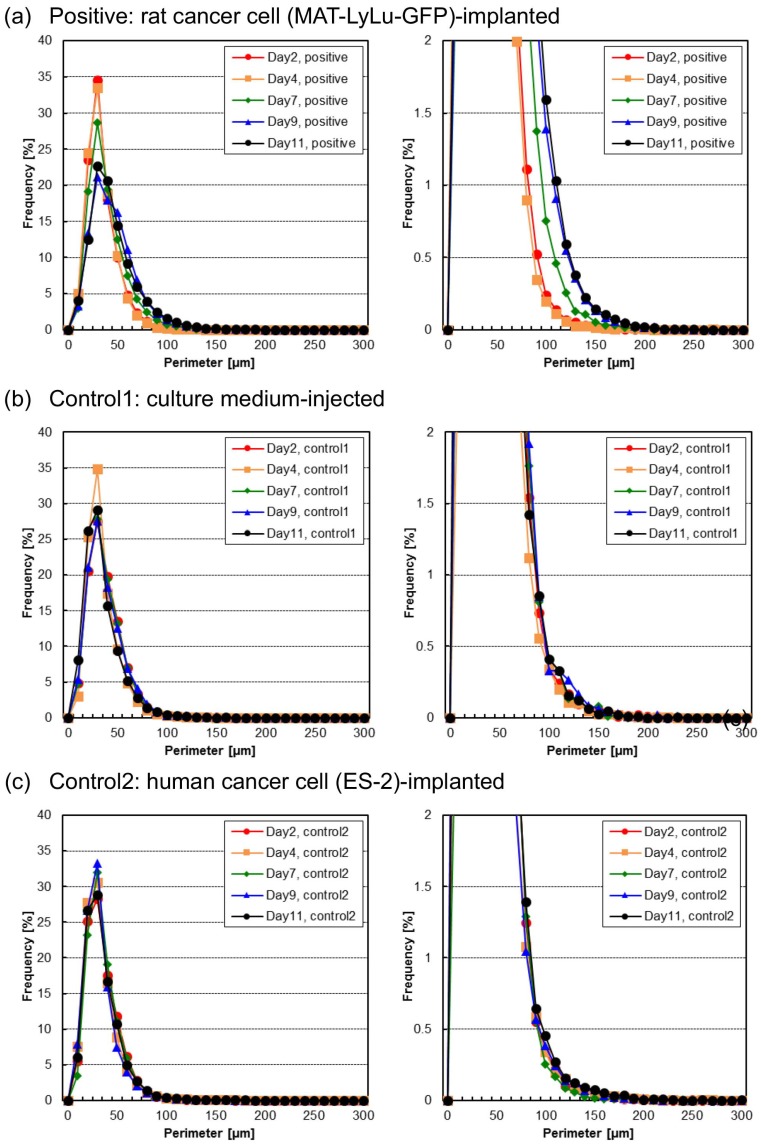
Perimeter length distribution of BF cell areas in positive (**a**), control 1 (**b**), and control 2 (**c**) blood of the same samples as in Figure 4. Each size distribution map indicates 2, 4, 7, 9, or 11 days after the implantation of rat cancer cells (**a**, positive), culture medium (**b**, control 1), or human cancer cells (**c**, control 2). Measurements were performed for six rats for positive blood and three for each control. The perimeter length distribution graphs on the right are magnified graphs of those on the left, to emphasize the details of the time-course changes of frequencies of larger perimeter lengths.

Figure 6 shows the distribution change of one of the other imaging biomarkers, the perimeter length distribution, of BF cell and cell cluster images in blood from Day 2 to Day 11 for positive (Figure 6a), control 1 (Figure 6b), and control 2 blood (Figure 6c).

As shown by the whole frequency distribution graphs on the left and the magnified graphs on the right in Figure 6, only the positive samples increased slightly from 100 μm to 200 μm over time. As such, the change of perimeter length can be used as an imaging biomarker to detect the change of blood cell contents during the progression of metastatic cancer. However, compared with the cell area distribution change in Figure 4, the change in perimeter length was not as sensitive to changes in the populations of cells of different sizes.

### 3.4. Time-Course Change of Aspect Ratio in Cells and Cell Clusters

**Figure 7:**
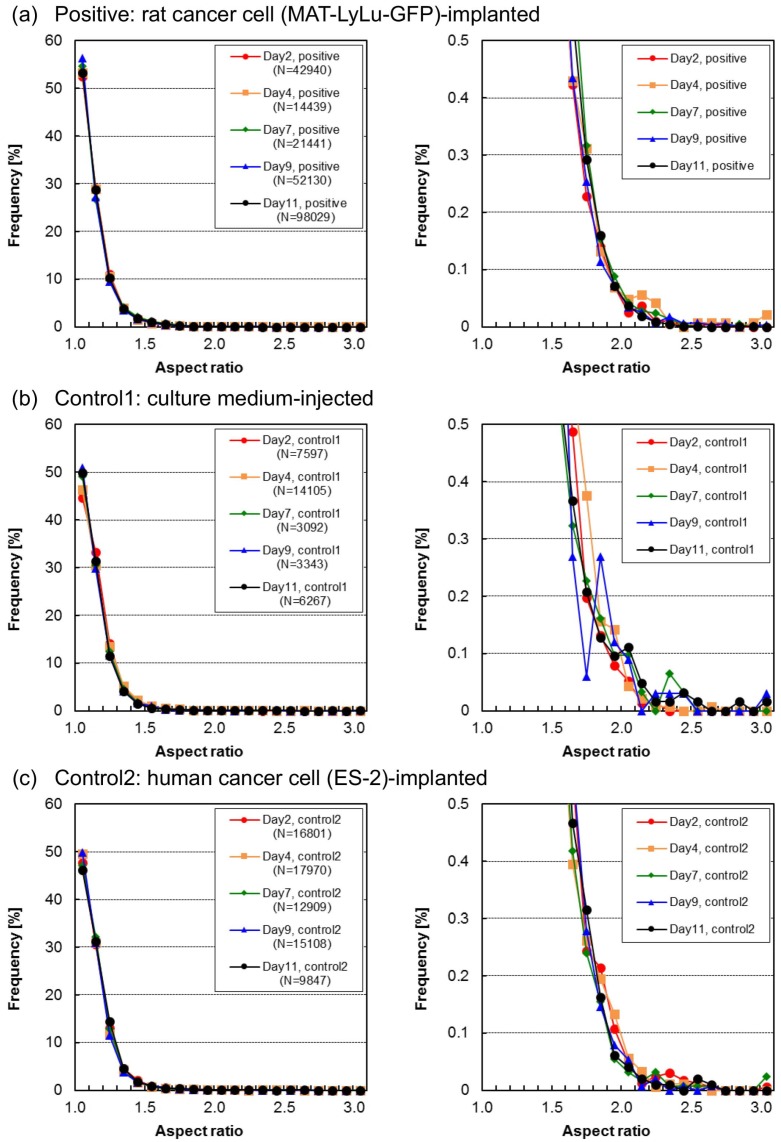
Aspect ratio distribution of BF cell areas in positive (**a**), control 1 (**b**), and control 2 (**c**) blood of the same samples as in Figure 4. Each size distribution map indicates 2, 4, 7, 9, or 11 days after the implantation of rat cancer cells (**a**, positive), culture medium (**b**, control 1), or human cancer cells (**c**, control 2). Measurements were performed for six rats for positive cases and three for the two types of control. The *x*-axis shows 0.2 intervals, while the *y*-axis (frequency) value at *x* = 1 represents the sample population percentage having an aspect ratio of 1 to 1.2. The graphs on the right are magnified versions of those on the left.

Figure 7 shows the aspect ratio distribution of BF cell and cell cluster images in blood over time from Day 2 to Day 11 for positive (Figure 7a), control 1 (Figure 7b), and control 2 blood (Figure 7c). As shown in these graphs, the ratio of the longer side to the shorter side was distributed from values representing round shapes (aspect ratio = 1) to rod-like shapes (aspect ratio ≫ 1), and more than 90% of all samples were within 1.4. Moreover, although the Control 1 sample fluctuated on Day 9 and Day 11, no significant tendencies of time-course changes were observed in both positive and control blood not only in samples with a lower aspect ratio but also in those with a higher aspect ratio of up to 3. This means that aspect ratio does not reflect the change of blood cell sizes and shapes over time after implantation.

**Figure 8:**
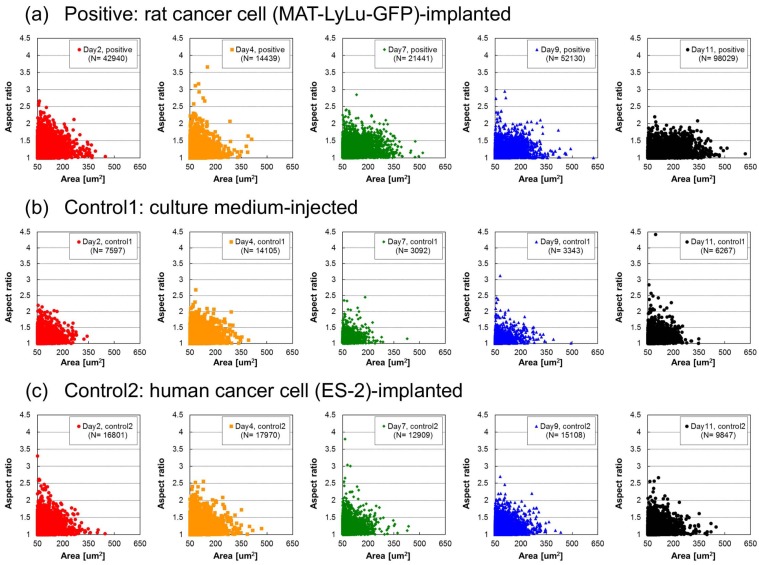
Cell size dependence of aspect ratio distribution of BF cell areas in positive (**a**), control 1 (**b**), and control 2 (**c**) blood of the same samples as in Figure 4. Each distribution graph is from 2, 4, 7, 9, or 11 days after the implantation of rat cancer cells (**a**, positive), culture medium (**b**, control 1), or human cancer cells (**c**, control 2). Measurements were performed for six rats for positive cases and three for controls. Each dot represents the cell size–aspect ratio of each sample.

The cell size dependence of the aspect ratio of BF cell and cell cluster images is replotted in Figure 8. In the graphs, data for each sample are plotted as dots, and their size–aspect ratio distribution is emphasized, whereas their population ratio is obscured because the magnitude of overlap of the dot plot is not properly reflected.

In all samples, the aspect ratio was generally larger for smaller samples, and vice versa. However, as it does not reflect the population ratio of those samples, we should regard these distributions as the possible maximum aspect ratio in their size distributions. This might also prove that the hydrodynamic focusing did not influence the shape of the cell clusters. If this focusing influences cluster shape formation, the aspect ratio in larger sample areas should have more of a tendency to represent a rod shape.

In positive samples (Figure 8a), time-course change of their distribution was observed. The small number of dots reflecting a larger aspect ratio for smaller samples was increased in Day 4 samples; however, they decreased gradually over time and finally, on Day 11, their maximum plots were less than 2.5. In larger samples, the maximum aspect ratio remained at smaller values, reflecting a similar tendency of the larger the size, the smaller the aspect ratio. Hence, the time-course change in aspect ratio shown in Figure 7a did not change even though the sample size distribution changed significantly over time. The results also indicate the tendency regarding the shapes of larger clusters in positive blood. The size of clusters increased over time and they showed a tendency to be round in shape.

In both of the two control samples (Figure 8a,b), their sample area–aspect ratio distributions were similar and no significant changes from the tendency of a larger size being associated with a smaller aspect ratio were observed. These results are consistent with the results in Figure 7a,b and can explain the lack of change over time.

### 3.5. Time-Course Changes of Cell Number, Nucleus Number, and Nucleus Area

**Figure 9:**
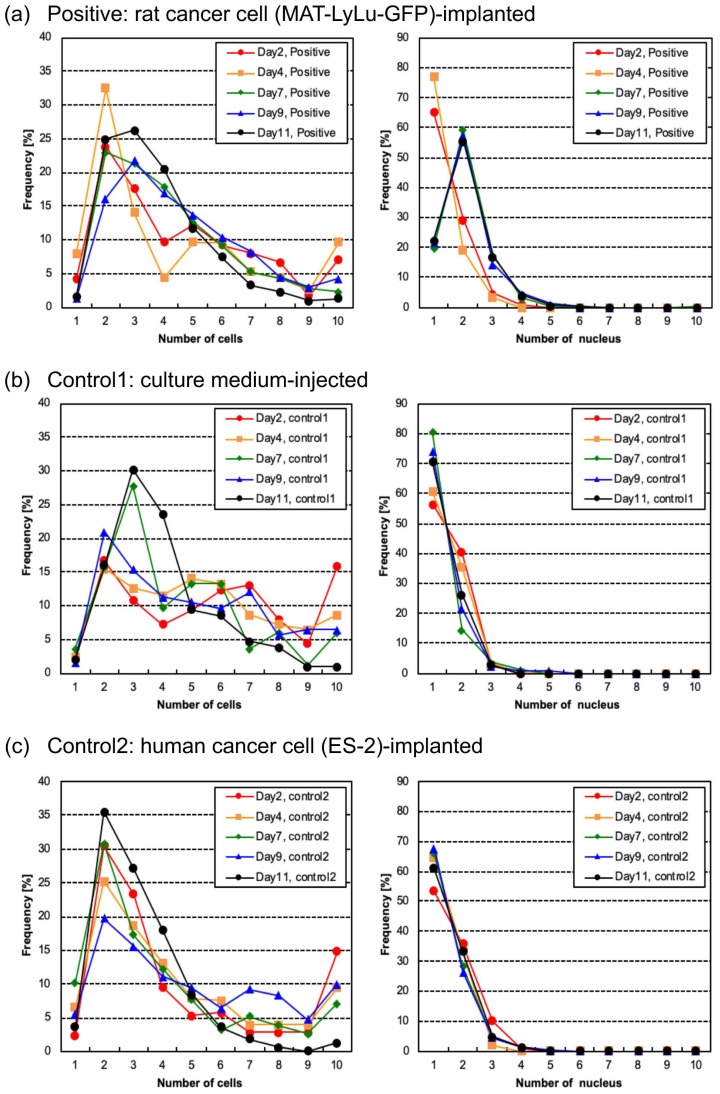
Time-course change of cell number and nucleus number distributions in positive (**a**), control 1 (**b**), and control 2 (**c**) blood of the same samples as in Figure 4. Each size distribution map indicates 2, 4, 7, 9, or 11 days after the implantation of rat cancer cells (**a**, positive), culture medium (**b**, control 1), or human cancer cells (**c**, control 2). Measurements were performed for six rats for positive cases and three for controls. Left graphs are the cell number distribution from BF images and right graphs are the nucleus number distribution from FL images.

Our cell sorter system can acquire other imaging biomarkers such as cell number from BF images, and nucleus number and nucleus area from FL images. Figure 9 shows the time-course changes of cell number (left graphs) and nucleus number (right graphs) in clusters in blood over time from Day 2 to Day 11 after implantation for positive (Figure 9a), control 1 (Figure 9b), and control 2 blood (Figure 9c).

Cell number distribution (left graphs in Figure 9) showed that the population ratios (frequencies) of cells were spread widely and did not show a particular tendency for change, but rather fluctuated over time from Day 2 to Day 11. In particular, in those graphs, there were two to three peaks obtained by estimating the cell number in BF images, which was not consistent with the cell size distributions in Figure 4. By comparison with raw BF images with these cell number counting results, debris attached to the cells was a major cause of those cell number distributions.

In contrast, the distributions of the numbers of nuclei (right graphs in Figure 9) were more stable and showed a clearer contrast between the positive samples and the two control samples. In the positive samples, single-nucleus samples were most common in the Day 2 and Day 4 samples, and their population peak increased to a level corresponding to two nuclei from Day 7 until Day 11. However, in control samples, their distributions of nucleus number did not change and remained similar over time.

These results provide some insight into cell number counting and nucleus number counting using imaging cell sorters. First, as described above, the cell number counting in BF images was not always consistent with the cell size distribution because of the attachment of debris. However, the distribution of the number of nuclei in FL images was not influenced by debris. This indicates the importance of simultaneous measurement of BF and FL images to avoid the influence of debris on cell number estimation, especially the importance of spatial information on the nucleus in FL images. This result also indicates that this cell number counting algorithm and procedure should be improved for CTC cluster measurement.

**Figure 10:**
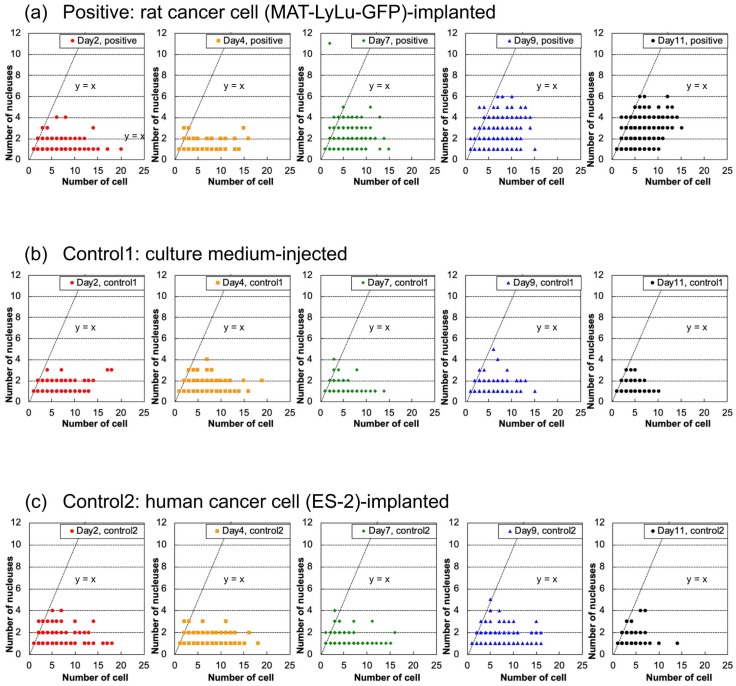
Time-course changes of cell number and nucleus number distribution in positive (**a**), control 1 (**b**), and control 2 (**c**) blood of the same samples as in Figure 4. The cell number–nucleus number distribution map indicates the results for 2, 4, 7, 9, and 11 days after the implantation of rat cancer cells (**a**, positive), culture medium (**b**, control 1), or human cancer cells (**c**, control 2). Measurements were performed for six rats for positive cases and three for controls. The data for each sample are plotted as a dot in the graphs. The dashed line corresponding to y=x represents the relationship of single cell with a single nucleus.

The correlation between cell number and nucleus number described in Figure 10 supports the explanation for the influence of debris on cell number counting. The same as in Figure 8, these graphs just present the maximum spatial distribution of samples and do not express their population frequencies because of overlap of their dot plotting.

The spatial distributions of dots representing samples in the two controls (Figure 10b,c) were similar and did not change significantly over time. However, the distribution of positive samples (Figure 10a) showed an increase in the maximum number of cells over time, and also showed an increase of larger clusters having a nucleus in each cell (y=x in graph) with up to six cells in the clusters, and an increase of samples with multiple nuclei (y≥x in graph) in three- to four-cell clusters.

Focusing on the spatial distribution of the dots representing samples less than y=x dashed lines (y≤x in the graph), this area represents the counted cell numbers being much larger than the numbers of nuclei; hence, it includes the particles with no nuclei, such as debris. For example, for single-nucleus samples, the maximum number of cells was 18 in Control 2 blood. In terms of the time-course changes in the distribution for both of the two positive controls, there were no significant increases over time; instead, rather gradual decreases in number occurred.

**Figure 11:**
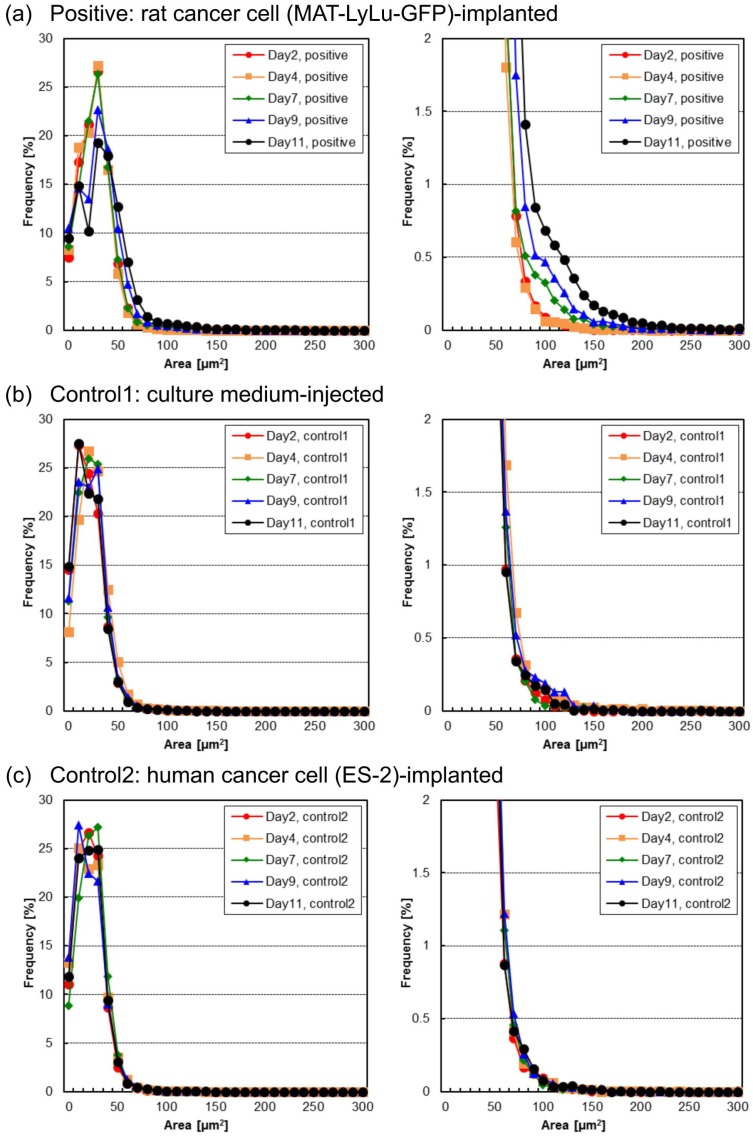
Time-course change of the distribution of total nucleus area in positive (**a**), control 1 (**b**), and control 2 (**c**) blood of the same samples as in Figure 4. Each nucleus area distribution map is from 2, 4, 7, 9, or 11 days after the implantation of rat cancer cells (**a**, positive), culture medium (**b**, control 1), or human cancer cells (**c**, control 2). Measurements were performed for six rats for positive cases and three for controls. The graphs on the right are magnified versions of those on the left.

The time-course changes of the distribution of the total area of nuclei in each sample in the positive and two control samples are also shown in Figure 11. As shown in the graphs on the right, their time-course changes were similar to and consistent with those of the BF sample size distributions (Figure 4), indicating that the total area of nuclei in single clusters in positive blood also correlated over time. In this analysis, as all of the areas of nuclei in single samples (cell or cluster) were calculated, the acquired plotting values represent the total number of nuclei of cells or clusters that have a single nucleus or multiple nuclei. When we can assume the typical size of nuclei in FL images, such as 20 μm^2^ from the value of the first peak in Figure 11, we can also estimate the number of cells in clusters from these nuclei more easily than in BF images, although there is still a risk of overestimating the cell numbers for cells with multiple nuclei.

The above results show the potential risks associated with counting cell numbers from BF images of cells given the uncertainty of image-based recognition of cells in clusters because of the existence of debris. In contrast, the usage of FL images for nucleus number and total area measurement showed more stable results. Considering all of the above conditions, the best way of measuring and identifying irregular cells in blood should be to use BF images for cell size and cell shape boundary data acquisition, and to use FL images for total nucleus number and area acquisition with position matching of BF cell shape boundary data. This approach would also provide a much simpler and hence much faster algorithm for CTC measurement than our former system. This simplification of the programming would also provide the opportunity to apply ultra-fast FPGA analysis for the identification of target samples in real-time cell sorting.

### 3.6. Correlation of Cell Size Distribution with Cell Number in Clusters

**Figure 12:**
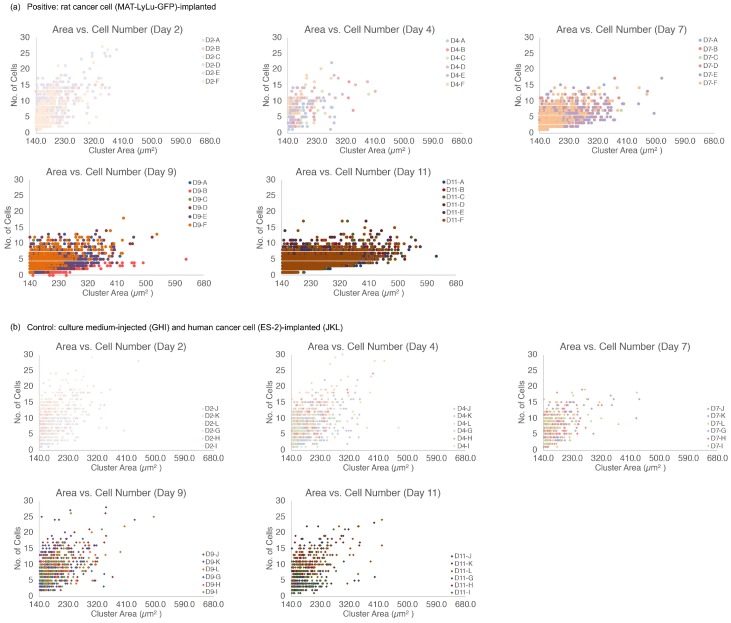
Relationship of cluster size and cell number within the clusters in positive blood (**a**), and combined results of control 1 and control 2 blood (**b**). Each graph shows the results 2, 4, 7, 9, or 11 days after the implantation of rat cancer cells (A–F, positive), culture medium (G–I, Control 1), or human cancer cells (J–L, Control 2).

In line with our results presented above, we examined the time-course change of the cell size distribution (using BF images) and cell numbers in those clusters (using FL images). Figure 12 shows the detailed results of analyzing the size distribution of cell clusters and their number of component cells within the clusters. The number of cells within a cluster was acquired from the number of nuclei in FL images within the cell contour shapes, which was acquired from BF images of those clusters. As shown in Figure 12a, the cluster size increased from Day 7 to Day 11, while the number of cells within the clusters was maintained or decreased, indicating that the cells in those clusters appeared to become larger. In contrast, the two types of control cells, as shown in Figure 12b, showed similar distributions over time and did not change, even on Day 11.

**Figure 13:**
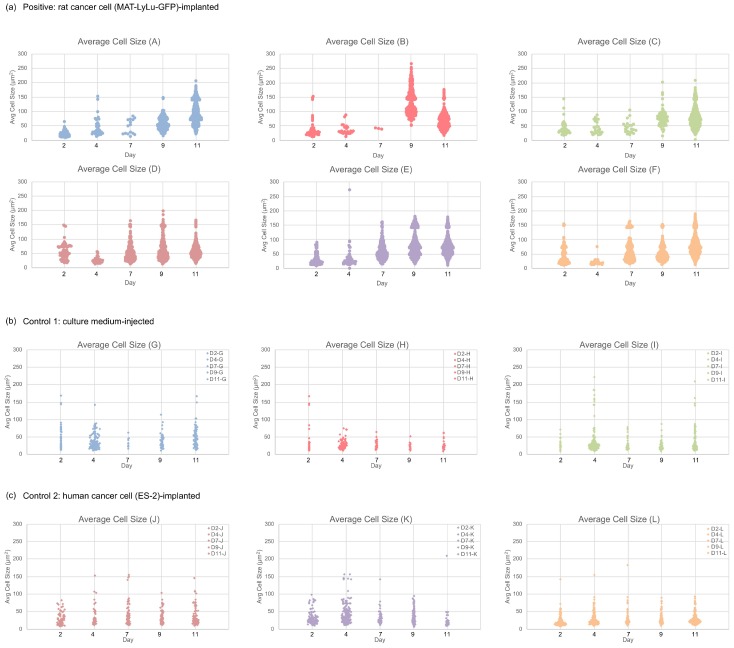
Time-course change of average size distribution of cells in positive (**a**), control 1 (**b**), and control 2 blood (**c**). Each value represents 2, 4, 7, 9, or 11 days after the implantation of rat cancer cells (**A**–**F**, six positive rats), culture medium (**G**–**I**, three rats of control 1), or human cancer cells (**J**–**L**, three rats of control 2). For each case, the *x*-axis represents the population at each average cell or cell cluster size on the *y*-axis.

To confirm the change in cell size in clusters, we replotted the average cell size distribution over time. Figure 13 shows the detailed analytical results of the time-course change of the size distribution of cells and their clusters in each individual sample rat. A tendency similar to that discussed for Figure 11 is presented in this figure. In positive blood samples (Figure 13a), their average size distributions changed in terms of both average maximum size and number in all six rats. However, in the average size distributions of control blood (Controls 1 and 2, in Figure 13b,c), no obvious change of their distribution was observed in either the three rats in Control 1 or the three rats in Control 2.

These results indicate the limitation of the conventional approach of simple cell size filtration for acquiring CTCs. As described above, even in healthy blood, cell clusters composed of single cells along with small debris might be present. Hence, to avoid a potential false-positive judgment in such cases, profiling of the size distribution map of blood cells as shown in Figure 13 should be performed.

**Figure 14:**
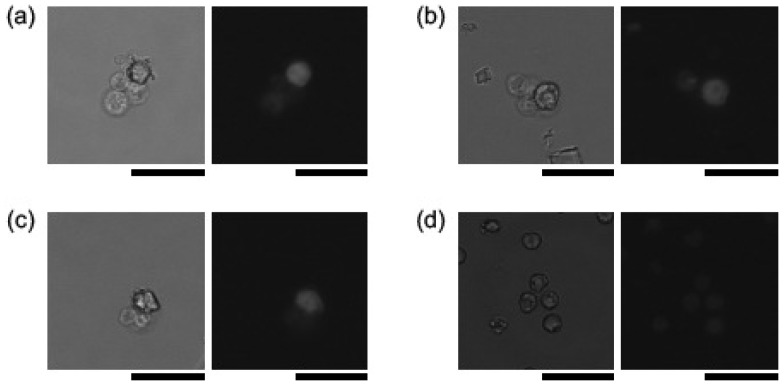
Three typical cell cluster images in positive cases (**a**–**c**) and a cell image in control 1 (**d**). Left, BF, and right, FL images. All FL images were taken with the same camera conditions. Bars, 50 μm.

**Figure 15:**
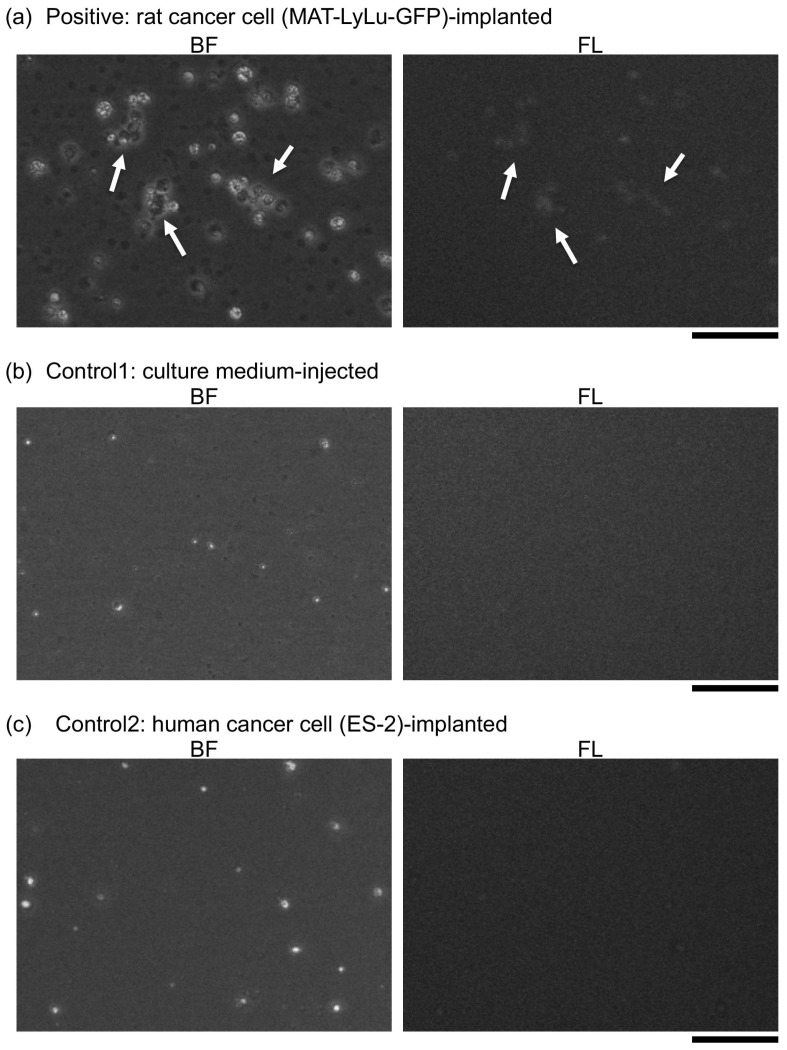
Photographs of cells for positive (**a**), control 1 (**b**), and control 2 blood (**c**) in wide areas. On the left are optical (BF) images of cells, while on the right are fluorescent (FL) images of corresponding positions. Arrowheads in (**a**) indicate cell clusters. Bars, 50 μm.

To confirm the obtained findings, cell clusters of 200 μm^2^ or larger were collected by our cell sorting system, and their GFP fluorescence was observed with a fluorescence optical microscope. It was confirmed that they were implanted cancer cells because of their green fluorescence, that is, CTCs with GFP fluorescence (Figure 14 and Figure 15). Therefore, we concluded that CTCs started to form large clusters from Day 7 and increased in number over time.

### 3.7. Ability and Limitation of On-Chip Imaging Cell Sorter for CTC Screening

In this study, as a follow-up examination of our previous work [20], to resolve the important issue of what happens in blood during progression in metastatic cancer, we examined the time-course change of CTC clusters of GFP-MAT-LyLu in positive rat blood over time after implantation. Their increase was detected by monitoring the time-course change of the distribution of imaging biomarkers as shown in Figure 4, Figure 5, Figure 6, Figure 7, Figure 8, Figure 9, Figure 10, Figure 11, Figure 12 and Figure 13.

The results in this study showed that CTC clusters clearly increased in number from Day 7. Those changes were monitored for several imaging biomarkers, such as cell size (Figure 4), perimeter length (Figure 6), nucleus number (Figure 9), and total nucleus area (Figure 11). In particular, the combined information of cell size and nucleus number (Figure 12) and average cell size distribution in clusters (Figure 13) gave us insights into how CTC cells and clusters grow during progression in metastatic cancer. These results indicate one of the abilities of imaging biomarkers for cancer diagnostics.

The results also provide some insight about the benefits and limitations of imaging biomarkers for CTC detection. For example, the index of the aspect ratio in BF images might not be suitable for identifying CTC clusters, especially for larger clusters. The results also recommend the use of cell size information combined with FL image information of nucleus number or total area because the existence of debris was sometimes the cause of larger-sized samples, even in the control sample blood.

Another finding in this study is that the numbers of CTCs and CTC clusters were greater than we expected. The size distribution analysis of whole blood samples provided important insights into the CTC population ratio in blood. Under the conventional understanding, the population of CTCs is very small and a blood sample on the order of mL is required for their detection. However, in our study, the population of irregular cells was greater than 1% even on Day 4, which means that around 1% of white blood cells were CTCs. If this assumption is correct, the required volume of blood for metastasis screening to acquire the size distribution of cells and for cell sorting should be only 10 to 100 μL, even for acquiring a sufficient number of target cells for the subsequent copy number analysis.

The limitation of our multi-imaging cell sorter is its time resolution. As the capturing of BF and FL images was simplified using a multi-image view unit for single CCD processing, image acquisition intervals were improved up to 10,000 fps. The software-based processing speed of simultaneous BF and FL image analysis including detailed BF cell number estimation was 200 fps in our system, whereas the simple FL nucleus number and area estimation procedure can be processed in the FPGA-based unit and accelerated to 5000–10,000 fps. Even if the hardware and software limitations were overcome, the limitation of FL intensity remained as a critical issue for the time resolution of the multi-imaging cell sorter. In our present light pathway set-up, weak fluorescence was hardly used for 5000-fps resolution. However, if the required volume of sample blood is 10 to 100 μL, the time required by the imaging cell sorter for analysis and cell sorting should be within a few hours, even using 200-fps mode.

In this paper, we present a novel antibody label-free approach for detecting irregular cells in blood using image-based recognition. In this work, morphometric parameters of cell clusters were measured and the validity of this approach for predictive CTC diagnostics was evaluated using microdroplet-sized blood samples.

## 4. Conclusions

In this study, a method for identifying irregular cells in blood by monitoring the distribution of imaging biomarkers in a 50-μL blood sample using on-chip multi-imaging flow cytometry was examined for monitoring the time-course change of their distributions during the progression of metastatic cancer. Using this method, significant increases in the number and size of CTC clusters were detected in blood of cancer cell-implanted rats, with them constituting more than 1% of whole white blood cells from four days after implantation of the GFP-MAT-LyLu cell line into rats. In contrast, no significant change was observed in control blood. These results indicate that the imaging biomarker detection of CTC clusters with a multi-imaging flow cytometry system is useful for the detection of irregular cells in blood, even with 50-μL blood samples, as a liquid biopsy for monitoring progression in metastatic cancer.

## References

[1] Cristofanilli M., Budd G.T., Ellis M.J., Stopeck A., Matera J., Miller M.C., Reuben J.M., Doyle G.V., Allard W.J., Terstappen L.W. (2004). Circulating tumor cells, disease progression, and survival in metastatic breast cancer. N. Engl. J. Med..

[2] Sethi N., Kang Y. (2011). Unravelling the complexity of metastasis—Molecular understanding and targeted therapies. Nat. Rev. Cancer.

[3] Yu M., Stott S., Toner M., Maheswaran S., Haber D.A. (2011). Circulating tumor cells: Approaches to isolation and characterization. J. Cell Biol..

[4] Davis J.A., Inglis D.W., Morton K.J., Lawrence D.A., Huang L.R., Chou S.Y., Sturm J.C., Austin R.H. (2006). Deterministic hydrodynamics: Taking blood apart. Proc. Natl. Acad. Sci. USA.

[5] Gascoyne P.R., Noshari J., Anderson T.J., Becker F.F. (2009). Isolation of rare cells from cell mixtures by dielectrophoresis. Electrophoresis.

[6] Nagrath S., Sequist L.V., Maheswaran S., Bell D.W., Irimia D., Ulkus L., Smith M.R., Kwak E.L., Digumarthy S., Muzikansky A. (2007). Isolation of rare circulating tumour cells in cancer patients by microchip technology. Nature.

[7] Stott S.L., Hsu C.H., Tsukrov D.I., Yu M., Miyamoto D.T., Waltman B.A., Rothenberg S.M., Shah A.M., Smas M.E., Korir G.K. (2010). Isolation of circulating tumor cells using a microvortex-generating herringbone-chip. Proc. Natl. Acad. Sci. USA.

[8] Zheng S., Lin H.K., Lu B., Williams A., Datar R., Cote R.J., Tai Y.C. (2011). 3D microfilter device for viable circulating tumor cell (CTC) enrichment from blood. Biomed. Microdevices.

[9] Budd G.T., Cristofanilli M., Ellis M.J., Stopeck A., Borden E., Miller M.C., Matera J., Repollet M., Doyle G.V., Terstappen L.W. (2006). Circulating tumor cells versus imaging–predicting overall survival in metastatic breast cancer. Clin. Cancer Res..

[10] Danila D.C., Heller G., Gignac G.A., Gonzalez-Espinoza R., Anand A., Tanaka E., Lilja H., Schwartz L., Larson S., Fleisher M. (2007). Circulating tumor cell number and prognosis in progressive castration-resistant prostate cancer. Clin. Cancer Res..

[11] Nieto M.A., Huang R.Y.J., Jackson R.A., Thiery J.P. (2016). EMT: 2016. Cell.

[12] Diepenbruck M., Christofori G. (2016). Epithelial–Mesenchymal Transition (EMT) and Metastasis: Yes, No, Maybe? CTC Metastasis. Curr. Opin. Cell Biol..

[13] Mong J., Tan M.H. (2018). Size-Based Enrichment Technologies for Non-cancerous Tumor-Derived Cells in Blood. Trends Biotechnol..

[14] Hao S.J., Wan Y., Xia Y.Q., Zou X., Zheng S.Y. (2018). Size-based separation methods of circulating tumor cells. Adv. Drug Deliv. Rev..

[15] Che J., Yu V., Garon E.B., Goldman J.W., Di Carlo D. (2017). Biophysical isolation and identification of circulating tumor cells. Lab Chip.

[16] Jan Y.J., Chen J.F., Zhu Y., Lu Y.T., Chen S.H., Chung H., Smalley M., Huang Y.W., Dong J., Chen L.C. (2018). NanoVelcro rare-cell assays for detection and characterization of circulating tumor cells. Adv. Drug Deliv. Rev..

[17] Huang X., Tang J., Hu L., Bian R., Liu M., Cao W., Zhang H. (2019). Arrayed microfluidic chip for detection of circulating tumor cells and evaluation of drug potency. Anal. Biochem..

[18] Takahashi K., Hattori A., Suzuki I., Ichiki T., Yasuda K. (2004). Non-destructive on-chip cell sorting system with real-time microscopic image processing. J. Nanobiotechnol..

[19] Hayashi M., Hattori A., Kim H., Terazono H., Kaneko T., Yasuda K. (2011). Fully automated on-chip imaging flow cytometry system with disposable contamination-free plastic re-cultivation chip. Int. J. Mol. Sci..

[20] Kim H., Terazono H., Nakamura Y., Sakai K., Hattori A., Odaka M., Girault M., Arao T., Nishio K., Miyagi Y. (2014). Development of on-chip multi-imaging flow cytometry for identification of imaging biomarkers of clustered circulating tumor cells. PLoS ONE.

[21] Hattori A., Kim H., Terazono H., Odaka M., Girault M., Matsuura K., Yasuda K. (2014). Identification of cells using morphological information of bright field/fluorescent multi-imaging flow cytometer images. Jpn. J. Appl. Phys..

[22] Vona G., Sabile A., Louha M., Sitruk V., Romana S., Schutze K., Capron F., Franco D., Pazzagli M., Vekemans M. (2000). Isolation by size of epithelial tumor cells—A new method for the immunomorphological and molecular characterization of circulating tumor cells. Am. J. Pathol..

[23] Desitter I., Guerrouahen B.S., Benali-Furet N., Wechsler J., Janne P.A., Kuang Y.A., Yanagita M., Wang L.L., Berkowitz J.A., Distel R.J. (2011). A new device for rapid isolation by size and characterization of rare circulating tumor cells. Anticancer Res..

[24] Hosokawa M., Kenmotsu H., Koh Y., Yoshino T., Yoshikawa T., Naito T., Takahashi T., Murakami H., Nakamura Y., Tsuya A. (2013). Size-based isolation of circulating tumor cells in lung cancer patients using a microcavity array system. PLoS ONE.

[25] Hosokawa M., Yoshikawa T., Negishi R., Yoshino T., Koh Y., Kenmotsu H., Naito T., Takahashi T., Yamamoto N., Kikuhara Y. (2013). Microcavity array system for size-based enrichment of circulating tumor cells from the blood of patients with small-cell lung cancer. Anal. Chem..

[26] Abdalla F., Boder J., Markus R., Hashmi H., Buhmeida A., Collan Y. (2009). Correlation of nuclear morphometry of breast cancer in histological sections with clinicopathological features and prognosis. Anticancer Res..

[27] Buhmeida A., Algars A., Ristamaki R., Collan Y., Syrjanen K., Pyrhonen S. (2006). Nuclear size as prognostic determinant in stage II and stage III colorectal adenocarcinoma. Anticancer Res..

[28] De Andrea C.E., Petrilli A.S., Jesus-Garcia R., Bleggi-Torres L.F., Alves M.T. (2011). Large and round tumor nuclei in osteosarcoma: Good clinical outcome. Int. J. Clin. Exp. Pathol..

[29] Deans G.T., Hamilton P.W., Watt P.C., Heatley M., Williamson K., Patterson C.C., Rowlands B.J., Parks G., Spence R. (1993). Morphometric analysis of colorectal cancer. Dis. Colon Rectum.

[30] Dundas S.A., Laing R.W., O’Cathain A., Seddon I., Slater D.N., Stephenson T.J., Underwood J.C. (1988). Feasibility of new prognostic classification for rectal cancer. J. Clin. Pathol..

[31] Meachem M.D., Burgess H.J., Davies J.L., Kidney B.A. (2012). Utility of nuclear morphometry in the cytologic evaluation of canine cutaneous soft tissue sarcomas. J. Vet. Diagn. Investig..

[32] Sokmen S., Sarioglu S., Fuzun M., Terzi C., Kupelioglu A., Aslan B. (2001). Prognostic significance of angiogenesis in rectal cancer: A morphometric investigation. Anticancer Res..

[33] Chou H.P., Spence C., Scherer A., Quake S. (1999). A microfabricated device for sizing and sorting DNA molecules. Proc. Natl. Acad. Sci. USA.

[34] Cheung K., Gawad S., Renaud P. (2005). Impedance spectroscopy flow cytometry: On-chip label-free cell differentiation. Cytom. Part A.

[35] Huh D., Gu W., Kamotani Y., Grotberg J.B., Takayama S. (2005). Microfluidics for flow cytometric analysis of cells and particles. Physiol. Meas..

[36] Cheung K.C., Di Berardino M., Schade-Kampmann G., Hebeisen M., Pierzchalski A., Bocsi J., Mittag A., Tárnok A. (2010). Microfluidic impedance-based flow cytometry. Cytom. Part A.

[37] Bow H., Pivkin I.V., Diez-Silva M., Goldfless S.J., Dao M., Niles J.C., Suresh S., Han J. (2011). A microfabricated deformability-based flow cytometer with application to malaria. Lab Chip.

[38] Karabacak N.M., Spuhler P.S., Fachin F., Lim E.J., Pai V., Ozkumur E., Martel J.M., Kojic N., Smith K., Chen P.I. (2014). Microfluidic, marker-free isolation of circulating tumor cells from blood samples. Nat. Protocols.

[39] Yu B.Y., Elbuken C., Shen C., Huissoon J.P., Ren C.L. (2018). An integrated microfluidic device for the sorting of yeast cells using image processing. Sci. Rep..

[40] Utharala R., Tseng Q., Furlong E.E., Merten C.A. (2018). A versatile, low-cost, multiway microfluidic sorter for droplets, cells, and embryos. Anal. Chem..

[41] Nitta N., Sugimura T., Isozaki A., Mikami H., Hiraki K., Sakuma S., Iino T., Arai F., Endo T., Fujiwaki Y. (2018). Intelligent image-activated cell sorting. Cell.

[42] Hattori A., Yasuda K. (2012). Extended depth of field optics for precise image analysis in microfluidic flow cytometry. Jpn. J. Appl. Phys..

[43] Kinosita K., Itoh H., Ishiwata S., Hirano K., Nishizaka T., Hayakawa T. (1991). Dual-view microscopy with a single camera: Real-time imaging of molecular orientations and calcium. J. Cell Biol..

[44] Hattori A., Kaneko T., Yasuda K. (2011). Improvement of particle alignment control and precise image acquisition for on-chip high-speed imaging cell sorter. Jpn. J. Appl. Phys..

[45] Tennant T.R., Kim H., Sokoloff M., Rinker-Schaeffer C.W. (2000). The Dunning model. Prostate.

[46] Yasuda K., Hattori A., Kim H., Terazono H., Hayashi M., Takei H., Kaneko T., Nomura F. (2013). Non-destructive on-chip imaging flow cell-sorting system for on-chip cellomics. Microfluid. Nanofluid..

